# Surface Plasmon Resonance as a Potential Diagnostic Tool for the Detection of CXC Chemokine Receptor 4 (CXCR4) on Extracellular Vesicles

**DOI:** 10.3390/bios16030174

**Published:** 2026-03-21

**Authors:** Kaat Verleye, Sam Noppen, Arnaud Boonen, Yagmur Yildizhan, Tom Van Loy, Cindy Heens, Frank Vanderhoydonc, Cláudio Pinheiro, Paula M. Pincela Lins, Annelies Bronckaers, An Hendrix, Johannes V. Swinnen, Dragana Spasic, Jeroen Lammertyn, Christophe Pannecouque, Dominique Schols

**Affiliations:** 1 Molecular, Structural and Translational Virology (Rega Institute), Department of Microbiology, Immunology and Transplantation, KU Leuven, 3000 Leuven, Belgium; sam.noppen@kuleuven.be (S.N.); yagmur.yildizhan@tss.ox.ac.uk (Y.Y.); tom.vanloy@kuleuven.be (T.V.L.); 2 Biosensors Group, Department of Biosystems, KU Leuven, 3000 Leuven, Belgium; dragana.spasic@kuleuven.be (D.S.); jeroen.lammertyn@kuleuven.be (J.L.); 3 Molecular Genetics and Therapeutics in Virology and Oncology (Rega Institute), Department of Microbiology, Immunology and Transplantation, KU Leuven, 3000 Leuven, Belgium; cindy.heens@kuleuven.be (C.H.); christophe.pannecouque@kuleuven.be (C.P.); 4 Laboratory of Lipid Metabolism and Cancer, Department of Oncology, KU Leuven, 3000 Leuven, Belgium; frank.vanderhoydonc@kuleuven.be (F.V.); j.swinnen@kuleuven.be (J.V.S.); 5 Laboratory of Experimental Cancer Research, Department of Human Structure and Repair, Ghent University, 9000 Ghent, Belgium; claudio.pinheiro@ugent.be (C.P.); an.hendrix@ugent.be (A.H.); 6 Department of Cardio and Organ Systems (COS), Biomedical Research Institute, Hasselt University, 3590 Diepenbeek, Belgium; paula.pincelalins@uhasselt.be (P.M.P.L.); annelies.bronckaers@uhasselt.be (A.B.)

**Keywords:** surface plasmon resonance, biosensor, extracellular vesicles, CXCR4, biomarker

## Abstract

This study leverages surface plasmon resonance (SPR) Biacore^TM^ technology to unveil the diagnostic potential of detecting CXCR4 on extracellular vesicles (EVs). Despite its recognized potential as a cancer biomarker, the presence of CXCR4 on EVs remains underexplored for diagnostic purposes. Using reference material (rEVs), a standardized label-free and real-time SPR biosensor is established to molecularly profile CXCR4-positive EVs. The binding interactions between immobilized antibodies and EVs isolated from different cancer cell lines revealed a unique SPR molecular fingerprint (SPR-MFP) consisting of varying expression levels of the CD9, CD63 and CD81 EV biomarkers, as well as CXCR4. There was a strong correlation between CXCR4 expression on the cellular membrane measured by flow cytometry (FCM) and the CXCR4 SPR signal of purified EVs, indicating that the chemokine receptor is actively transferred to the extracellular space. The Biacore^TM^ biosensor is able to directly detect and molecularly profile EVs in buffer and spiked in cell culture supernatant supplemented with 10% EV-depleted serum. Altogether, our findings illuminate the potential of SPR Biacore^TM^ technology in EV-related research as well as reveal the diagnostic potential of EV-associated CXCR4, offering valuable insights and paving the way for medical applications in diseases associated with aberrant CXCR4 expression.

## 1. Introduction

In recent years, extracellular vesicles (EVs) have gained increasing interest as promising biomarkers and drug delivery vehicles. These nanoparticles, ranging from 40 to 1000 nm in size, are surrounded by a phospholipid bilayer; secreted by all cell types; and carry proteins, lipids and nucleic acids, reflecting their cell of origin and production mechanism [1,2]. EVs are important in both physiological processes and pathogenesis of various human diseases, such as cancer, cardiovascular disease and viral infections [3,4]. As biomarkers, EVs offer a valuable, yet underexplored, tool for cancer detection [5]. Several detection technologies have been used to characterize EVs, including ELISA, PCR, Western blot, flow cytometry, single-particle tracking, electron microscopy and surface plasmon resonance (SPR). Although the latter offers several advantages, it remains an underused technique in the profiling of EVs compared to the other detection technologies [6,7,8]. For instance, SPR is extremely sensitive to molecular binding events within 150 nm from the surface, which makes it an ideal tool for detecting (small) EVs. Moreover, the technique enables label-free, real-time analysis of molecular interactions on a gold surface and consumes relatively small amounts of a sample and has a high throughput, therefore representing an ideal candidate to be utilized for laboratory-based diagnostic purposes.

Among the various SPR platforms currently available, Biacore^TM^—one of the first commercial SPR biosensors—has been widely used in SPR-based biosensing studies. In a typical Biacore^TM^ experiment that detects EVs in a direct and label-free manner, EV-specific antibodies or receptors are captured on a biosensor chip, and they interact with (isolated) EVs free in solution. After binding of EVs, the biosensor chip can be regenerated in order to capture fresh antibodies and/or EVs. Although previous SPR studies have successfully detected cancer-derived EVs using molecular profiling approaches, most articles lack standardized reporting of regeneration procedures and limits of detection (LOD) [9,10,11,12,13,14].

There are diverse applications that SPR can offer to the EV field, both for research and as a diagnostic tool. In this study, we used SPR technology for the detection of C-X-C motif chemokine receptor type 4 (CXCR4) on purified EVs from a cell culture medium (CCM). Chemokine receptors belong to the family of G protein-coupled receptors (GPCRs), also known as 7-transmembrane receptors [15]. The activity of chemokines and their receptors is regulated at different levels [16]. Recently it was shown that GPCRs are involved in the biogenesis, homing and uptake of EVs, and conversely that EVs can attenuate GPCR signaling through receptor down modulation and act as circulating decoy receptors [17]. Among 20 endogenous chemokine receptors in humans and mice [18], CXCR4 excels with its functional roles in physiological and pathological conditions and is highly expressed in various cell types, including lymphocytes; endothelial, epithelial and hematopoietic stem cells; stromal fibroblasts; and cancer cells [19]. Over the years, CXCR4 has been associated with several diseases, including more than 23 types of cancer [20,21], neurodegeneration [22,23], autoimmunity [15,24], and immunodeficiencies [25]. Remarkably, since its discovery in 1996, CXCR4 has been revealed to function as one of the co-receptors for the entry of T-tropic human immunodeficiency virus-1 (HIV-1) into CD4^+^ cells during the later stages of HIV infection [26]. Interestingly, CXCR4 present on EVs can be horizontally transferred to CXCR4-null cells rendering the acceptor cells susceptible to infection with HIV [27]. Aside from being implicated in HIV-1 infection, CXCR4 is overexpressed on various tumor-derived EVs, and hence could play an important role as a biomarker in cancer diagnostics. Wang et al. reported that CXCR4 EV proteins are associated with tumor recurrence or distant organ metastasis in breast cancer patients [28]. Another study demonstrated the expression level of CXCR4 in serum EVs from glioma patients at different stages, with stage III and stage IV patients expressing higher levels of CXCR4 as compared to stage I and II patients [29]. Among the other pro-angiogenic factors present in EVs from glioblastoma cells, CXCR4 was found to be a key regulator of angiogenesis, and hence facilitates tumorigenesis [30]. An interesting study utilizing machine learning showed that serum-derived small EVs exhibit a significantly higher CXCR4 level in advanced-stage non-small-cell lung cancer patients compared to early-stage patients and healthy donors [31]. Kalinkovich et al. found that circulating EVs with functional CXCR4 are involved in the progression of acute myelogenous leukemia (AML) and can be used as a biomarker for AML [32]. The transfer of EV-associated CXCR4 promotes hepatocarcinoma cell migration, invasion and lymphangiogenesis [33]. Emerging reports are demonstrating the involvement of CXCR4-expressed EVs in a range of cancer types [34,35,36,37]. Additionally, CXCR4 plays a well-established role in the homing of mesenchymal stem cells, guiding migration towards sites of injury [38,39].

Despite these findings, CXCR4 has not yet been commonly recognized or applied as an EV biomarker for cancer diagnostic purposes or therapeutic monitoring. Thus, this study leverages SPR technology to study CXCR4-positive EVs. In this context, recombinant EVs (rEVs) were used to develop and standardize a direct and label-free SPR biosensor on a Biacore^TM^ T200 [40,41]. The LOD, efficiency, specificity and reproducibility of the assay were assessed using CD9, CD63 and CD81 EV biomarkers, as well as CXCR4. Flow cytometry (FCM) was employed to select seven cancer cell lines, expressing low and high amounts of CXCR4. The EVs were subsequently isolated from these cell lines with size exclusion chromatography (SEC), previously optimized using rEVs. Presence of isolated EVs was confirmed using transmission electron microscopy (TEM), while the EVs were quantified with nanoparticle tracking analysis (NTA). Finally, the expression of CD9, CD63, CD81 and CXCR4 on the isolated EVs from seven cancer cell lines was analyzed using the SPR biosensor and revealed a unique SPR molecular fingerprint (SPR-MFP). Interestingly, there was a strong correlation (r = 0.7960) between the CXCR4 EV SPR signal and CXCR4 expression on the parental cells measured by FCM. This might indicate that EV-associated CXCR4 can be used as a biomarker for CXCR4-associated pathologies. A single-particle interferometric reflectance imaging sensor (SP-IRIS) was used to benchmark the SPR results and to demonstrate the colocalization of CXCR4 with CD9, CD63 and CD81 at a single EV level directly in culture supernatant. In addition, Biacore^TM^ technology was able to detect and discriminate between the EVs from HEK293 and HEK293.CXCR4 cells spiked in a crude mixture (i.e., cell culture supernatant supplemented with 10% EV-depleted serum) based on the specific SPR-MFP. This paves the way for a new SPR biosensor to be explored as a potential diagnostic tool.

## 2. Materials and Methods

### 2.1. Reagents and Antibodies

Dulbecco’s minimal essential medium (DMEM), Roswell Park Memorial Institute (RPMI) 1640, 0.25% trypsin/EDTA, 2-(4-(2-hydroxyethyl)-1-piperazinyl)ethanesulfonic acid (HEPES), fetal bovine serum (FBS, heat-inactivated), sodium pyruvate, geneticin, gentamicin, Opti-MEM, and Dulbecco’s phosphate-buffered saline (PBS) were obtained from Thermo Fisher Scientific (Waltham, MA, USA). EV-depleted (ED) FBS was purchased from System Biosciences (Palo Alto, CA, USA). Bovine serum albumin (BSA) was obtained from Cell Signaling Technology (Beverly, MA, USA).

The reagents used during Biacore^TM^ SPR experiments, HEPES and sodium chloride (NaCl), were obtained from Carl Roth (Karlsruhe, Germany), while 1-ethyl-3-(3-dimethylaminopropyl)carbodiimide (EDC), N-hydroxysuccinimide (NHS), 10 mM sodium acetate pH 5.0, 1 M ethanolamine pH 8.0, capture kits and regeneration solutions were obtained from Cytiva (Uppsala, Sweden). Triton X-100, isopropanol and sodium hydroxide (NaOH) were obtained from Merck (Darmstadt, Germany). All SPR buffers were prepared in 50 mM NaOH precleaned glassware using ultra-pure water from a Milli-Q purification system (Merck) and passed through a 0.2 µm PES membrane filter (Thermo Fisher Scientific).

The following antibodies against EV tetraspanins were used: anti-CD9 (Cat. no: 10626D, clone: TS9) obtained from Thermo Fisher Scientific; anti-CD63 (Cat. no: EX204, clone: CGS82X) obtained from Cell Guidance Systems (Cambridge, UK); and anti-CD81 (Cat. no: 555675, clone: JS-81) obtained from BD biosciences (San Diego, CA, USA). The following BD biosciences antibodies against CXCR4 (clone 12G5) were used: 12G5-APC (Allophycocyanin, fluorescent dye, Cat. no: 555976), 12G5-PE (phycoerythrin, fluorescent dye, Cat. No: 555974) and 12G5 (Cat. no: 555972). The following BD biosciences antibodies against the isotypes were used: IgG1 (Cat. no: 555746), IgG2a (Cat. no: 550339), IgG2a-APC (Cat. no: 555576) and IgG2a-PE (Cat. No: 555743). All antibody concentrations are indicated in the following sections.

The Alliance HIV-1 p24 Ag ELISA kit (cat. No: NEK050B001KT) was purchased from Revvity (Waltham, MA, USA).

The following cell lines were used: human embryonic kidney 293 cells (HEK293), HEK293 stably transfected with hCXCR4 (HEK293.CXCR4), U87 malignant glioblastoma cells (U87), U87 stably transfected with hCXCR4 (U87.CXCR4), human T cell acute lymphoblastic leukemia MOLT-4 cells (MOLT-4), Michigan Cancer Foundation-7 cells (MCF-7) and adult T cell leukemia MT-4 cells (MT-4). The corresponding CCM and source are mentioned in Table S1.

### 2.2. rEV Generation and Isolation

Generation and isolation of rEVs was performed as previously described [39]. In short, the plasmid pMET7-gag-EGFP was purified from DH10B *E. coli* via the PC2000 nucleobond kit (Cat. No: MN 740576, Macherey-Nagel, Düren, Germany) according to the manufacturer’s instructions. For rEV generation, HEK293T cells were seeded in Falcon cell culture Multi-Flasks (Cat. No: 353144, Corning incorporated, Corning, NY, USA) and transiently transfected at 70–80% confluency using 25 kDa linear polyethyleneimine (PEI) (Cat. No: 23966, Polysciences, Niles, IL, USA) in a PEI:DNA ratio of 5:1 with a final concentration of 1 µg DNA/mL culture medium in a total volume of 120 mL per Multi-Flask. After 48 h, the cells were washed three times using Opti-MEM followed by 24 h incubation in Opti-MEM supplemented with 100 IU/mL penicillin and 100 mg/mL streptomycin at 37 °C and 10% CO_2_. The resulting conditioned medium (CM) was then collected and centrifuged for 10 min at 300× *g* and 4 °C to remove the dead, detached cells. Next, 0.45 µm cellulose acetate filtration (Cat. No: A35999, NovoLab, Geraardsbergen, Belgium) was implemented to remove the larger particles. The filtrated medium was concentrated to 1 mL using a Centricon Plus-70 centrifugal filter device with a 10 kDa nominal molecular weight limit (Cat. No: UFC701008, Merck). The resulting concentrated CM was utilized for the OptiPrep velocity gradients (OVGs).

The OVGs were prepared by layering 11 iodixanol solutions (Cat. No: AXI-1114542, Axis-Shield, Oslo, NO) of 1.3 mL ranging from 18 to 6% with 1.2% decrements from the bottom to the top in a 16.8 mL open top polyallomer tube (Cat. No: 337986, Beckman Coulter, Indianapolis, IN, USA). One milliliter of concentrated CM was overlaid on top of the gradient that was then centrifuged for 1 h and 56 min at 186,700× *g* at 4 °C (SW 32.1 Ti rotor, Beckman Coulter). All OVGs were assembled using a biomek 4000 automated workstation (Beckman Coulter). Solutions of 18–6% iodixanol were made by mixing appropriate amounts of PBS with a 60% (*w*/*v*) iodixanol stock solution. Following centrifugation, gradient fractions of 1 mL were collected from top to bottom using the biomek 4000 automated workstation. Fractions 10–13, corresponding to buoyant density of 1.076–1.088 g/mL, were collected, pooled and diluted to 16 mL in PBS and centrifuged for 3 h at 100,000× *g* at 4 °C (SW 32.1 Ti rotor, Beckman Coulter). The resulting pellet was resuspended in PBS and stored at −80 °C until further use.

### 2.3. Label-Free EV Detection Bioassay Development with Biacore^TM^ SPR

**Figure 1:**
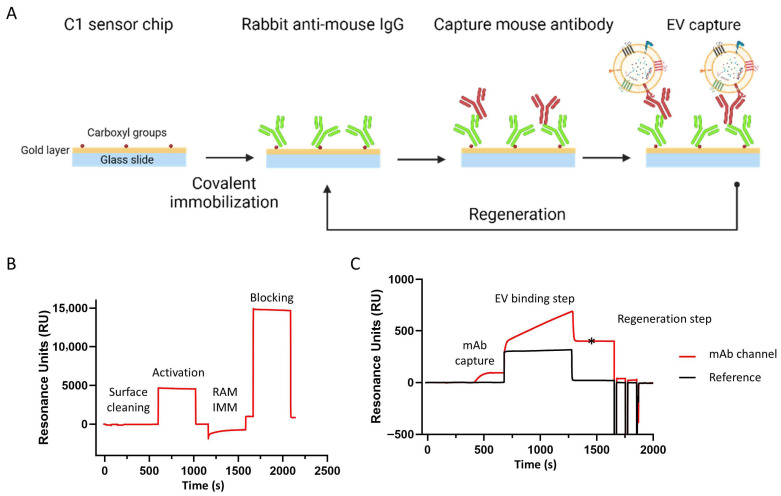
(**A**) Schematic demonstration of label-free EV capture bioassay using C1 sensor chips of Biacore^TM^ SPR technology. Created with BioRender.com (not drawn to scale). SPR sensorgram representing (**B**) activation of C1 sensor chip followed by RAM immobilization and blocking steps and (**C**) mouse monoclonal antibody capture followed by EV binding and regeneration step. Reference refers to sample without addition of antibodies. Asterisk indicates time point at which amount of bound EVs was measured.

Label-free EV detection was performed using a Biacore^TM^ T200 (Cytiva) SPR system. A C1 sensor chip (carboxymethylated, matrix-free surface for covalent immobilization obtained from Cytiva) was used in combination with HBS buffer (0.01 M HEPES pH 7.4, 0.15 M NaCl) for the analysis of the SEC fraction 5 obtained during the EV isolation process. A mouse antibody capture kit (Cytiva) was used to capture mouse monoclonal antibodies onto the chip (Figure 1A). The chip was first conditioned using 2 injections, each lasting 60 s, of 0.1 M glycine-NaOH and 0.3% Triton X-100 at pH 12 for 30 µL/min (Figure 1B). Next, the chip was activated with a 7 min long injection of 0.2 M EDC/0.05 M NHS at 10 µL/min. Afterwards, the rabbit anti-mouse (RAM) IgG antibody, diluted to 30 µg/mL in 10 mM sodium acetate buffer pH 5, was injected over the chip for 7 min at 10 µL/min. Finally, the chip was deactivated with a 7 min long injection of 1.0 M ethanolamine pH 8 solution. The buffer was changed to an HBS-BSA buffer with 1 mg/mL BSA. Once the chip was functionalized with RAM IgG antibody, different EV biomarker mouse antibodies (anti-CD9, anti-CD63 and anti-CD81), a 12G5 anti-CXCR4 monoclonal antibody and 2 isotype controls (IgG1 and IgG2a) were captured onto the chip at different concentrations, reaching approximately 100 RU for each antibody (Figure 1C). A reference flow without captured mouse antibodies was used as a control for non-specific binding and refractive index changes. Following this, SEC fraction 5 was diluted 1:10 in running buffer and injected over the different antibodies for 600 s at 2 µL/min. After each binding instance, the chip was regenerated with glycine-HCl pH 1.7 and 0.5% Triton X-100. An extra washing step with 20% isopropanol in 40 mM NaOH was used to keep the flow system clean. Sensor chips could be used at least 100 times without any significant loss of binding capacity. Several buffer blanks were used for double referencing. The RUs at 100 s after injection for each sample were compiled to analyze the relative amount of EVs in each SEC sample.

Calibration curves were made following the same procedure and using the same antibodies (anti-CD9, anti-CD63, anti-CD81 and 12G5 anti-CXCR4). rEVs were injected using a concentration range of a threefold serial dilution from 1 × 10^7^ to 3 × 10^9^ particles/mL.

For EV detection in the crude mixtures, the EVs prepared from HEK293 and HEK293.CXCR4 cells were spiked in DMEM supplemented with 10% 0.2 µm filtered ED-FBS. For this experiment, the SEC fraction with the highest EV concentration measured with NTA was selected. The EV suspensions were diluted 1:6 in the medium, and the resulting sample was further diluted 3:2 in HBS-BSA buffer before injection into the Biacore^TM^ system (thus having a final dilution of 1:10), resulting in a final concentration of 1.22 × 10^9^ particles/mL for HEK293 EVs and 3.53 × 10^9^ particles/mL for HEK293.CXCR4 EVs. Analysis was performed using a C1 chip following the same procedure as described previously.

### 2.4. Flow Cytometry (FCM) Analysis

To perform FCM analysis, all cell lines, with exception of the suspension cell lines MT-4 and MOLT-4, were digested using 0.25% trypsin/EDTA and resuspended in their appropriate CCM. This was followed by 2 h of incubation at 37 °C to reconstitute receptor expression on the cell surface after possible reduction in receptor levels due to trypsinization. For each cell line, 2 × 10^5^ cells were washed twice with, and then resuspended in PBS containing 2% FBS. Afterwards, they were treated with either 12G5-PE (for U87 and U87.CXCR4 cells) or 12G5-APC (all other cell lines) antibodies and an IgG2a-PE or IgG2a-APC isotype control at room temperature for 60 min. Untreated cells were also used as an additional control. Cell suspensions were fixed in 1% paraformaldehyde (Merck) in PBS and analyzed with the FACSCelesta^TM^ flow cytometer (BD Biosciences).

### 2.5. Isolation of EVs from Different Cell Lines

For adherent cell lines, 10^6^ cells were seeded in a T75 culture flask (two flasks in total) in 15 mL of their respective CCM and incubated at 37 °C in 5% CO_2_. After 72 h, the medium was aspirated, the cells were washed, and the medium replaced by 15 mL of DMEM supplemented with 0.5% ED-FBS. For suspension cells, 0.2 × 10^6^ cells/mL were seeded and cultured for 72 h. Subsequently, 1.2 × 10^6^ cells/mL were centrifuged, washed and resuspended in 40 mL of RPMI containing 0.5% ED-FBS. After 24 h incubation in CCM with ED-FBS, the supernatant was collected for EV isolation, while the cells were counted, and their viability was assessed.

The collected supernatant was centrifuged (Thermo Fisher Scientific Sorvall^TM^ ST 40 Centrifuge Series) at 300× *g* at 4 °C for 6 min (Figure S1), filtered through 0.45 µm SFCA membrane filter (syringe) (Corning incorporated), and concentrated with 2 Amicon Ultra-15 centrifugal regenerated cellulose filter units (Merck) at 4 °C, 3000× *g* for 30 min. The collected concentrate was again filtered through a 0.2 µm PES membrane filter (Pall life sciences, Hoegaarden, Belgium). The SEC was performed using an in-house created column (Cytiva) containing 10 mL of small porous polymer beads (Sepharose CL-2B (Merck)). First, the column was washed with 30 mL PBS. Then, filtered CCM was added to the column, while adding PBS on top to reach 1 mL (650 µL CCM + 350 µL PBS) to collect the first fraction. After adding 1 mL of PBS, the rest of the 11 fractions were collected. BSA was added into each SEC fraction to reach a final concentration of 1 mg/mL, and the fractions were concentrated with Amicon 2 mL regenerated cellulose filters (Merck) at 4 °C, 3000× *g* for 30 min. Finally, Amicon 2 mL filters containing 110 µL solution were reverse-spun for 2 min at 4 °C, 1000× *g* and stored in protein LoBind tubes (Eppendorf, Aarschot, Belgium) at −80 °C for further use.

### 2.6. Nanoparticle Tracking Analysis (NTA)

NTA was performed using SEC fraction 5 from each cell line diluted in PBS buffer to a final volume of 800 μL. Measurements were conducted with a NanoSight LM10 (Malvern Instruments, Worcestershire, UK) equipped with a 405 nm laser. An sCMOS camera with varying shutter lengths was used for recording. The detection threshold was set at 4. The shutter settings and camera gain were respectively 450 and 250. Timelapses of 30 s were captured among six videos. The data was analyzed using NanoSight NTA analytical software (version 2.3, NanoSight Ltd., Wiltshire, UK).

### 2.7. Transmission Electron Microscopy (TEM)

To prepare the samples for TEM, 3 µL of the purified EVs from each cell line was carefully placed onto an ultrathin carbon film supported by a Lacey carbon copper grid (Ted Pella, Redding, CA, USA) for 60 s and blotted dry with filter paper. The samples were washed three times with 10 µL of PBS for 30 s. The samples were stained by successively applying 3 µL of 2% uranyl acetate twice for 30 s. TEM images were captured using a JEOL-1400 FLASH (JEOL, Peabody, MA, USA) microscope operating at 80 kV, equipped with a Xarosa camera (GE), and images were collected from at least 10 different areas.

### 2.8. HIV-1 p24 Ag ELISA of rEVs

Analysis of p24 HIV-1 Ag on rEVs was performed according to the Alliance HIV-1 p24 ELISA assay protocol. The samples, standard curve and negative control were all diluted in PBS. Before transferring to the antibody-coated microplate, Triton X-100 was added. After a 2 h incubation step at 37 °C during which the p24-antigens could bind to the surface of the plate, all the wells were washed five times. A second 1 h incubation step at 37 °C was performed with detector antibody, followed by a five-cycle wash step. After adding streptavidin-HRP, the plate was incubated for 30 min at room temperature. A final washing cycle was then carried out. Since o-phenylenediamine dihydrochloride is light-sensitive, the final 30 min incubation was performed in the dark. A yellow color developed, and the reaction was stopped prior to reading absorbance on a Tecan Spark plate reader (Männedorf, Schweiz).

### 2.9. SP-IRIS Sandwich Bioassay for EV Detection

ExoView R200 (Unchained Labs, Pleasanton, CA, USA) was applied to visually demonstrate CXCR4 expression on the EVs from HEK293 and HEK293.CXCR4 directly in their cell culture supernatant and SEC fraction 5. ExoView microarray chips functionalized with antibodies against CD9, CD63, CD81 and the isotype control were obtained from Unchained Labs (details of surface functionalization unknown). First, they were pre-scanned to generate baseline measurements. To prevent chip oversaturation, cell culture supernatant and SEC fraction 5 were diluted 1:10 in incubation solution (Unchained Labs), and 50 µL was loaded onto ExoView chips to incubate overnight at room temperature. To wash the unbound EVs, the ExoView chips were submerged in 1000 µL Solution A (Unchained Labs) for 3 min at 500 rpm on an orbital shaker. This was followed by three washes, each involving aspiration of 750 µL and replacement with fresh solution A. Afterwards, the captured EVs were fluorescently stained with anti-CXCR4 antibody (12G5-APC) following the manufacturers’ instructions. The 12G5-APC antibody (0.4 µg/mL) was prepared in 300 µL Blocking Solution (Unchained Labs) and vortexed for 5 s. Then, 250 µL was added to each ExoView chip and incubated for 1 h at 500 rpm, with an aluminum foil covering to prevent fluorophore bleaching. The unbound antibodies were removed by three sequential instances of washing with 500 µL of Solution A for 3 min at 500 rpm, followed by the same procedure with solution B (Unchained Labs). Finally, 750 µL of solution B was aspirated and replaced with 750 µL of Milli-Q water. The chips were dried by placing them in a Petri dish at a 45 degree angle, allowing for surface tension to remove excess water. Residual moisture was absorbed using paper tissue before transferring the chips to the sample stage for fluorescent and interferometric imaging.

### 2.10. Data Analysis

The calibration curves in Section 3.1 were fitted using simple linear regression from Graphpad Prism software version 10.6. The LOD was calculated using the equation (3.3*σ)/S = LOD, with σ being the standard deviation of the curve and S the slope of the curve. The coefficient of variation (CV) was calculated by dividing the standard deviation by the mean and multiplying by 100%. The FCM data in Section 3.3 was processed using FLOWJO version 10.9. The fold change was calculated using the mean fluorescent intensity (MFI) in the equation (MFI_positive_/MFI_negative_). The negative control was used for MFI_negative_. The SPR data presented in Section 3.4 and Section 3.6 were normalized by transforming the raw data based on the equation Yf = Yi/K. Yf is the normalized Biacore^TM^ SPR signal, Yi is the initial Biacore^TM^ SPR signal, and K is the normalization coefficient calculated separately for each EV subtype. The K values are the EV concentration in 10^9^ particles/mL measured by NTA analysis. The correlation data presented in Section 3.4 were fitted using simple linear regression from Graphpad Prism software version 10.6. The data was analyzed using the Pearson correlation. The ExoView data presented in Section 3.5 was analyzed to assess the statistical difference between different the experimental conditions. Mixed effects analysis with Tukey’s multiple comparison test was used. The averages denoted with the same letter indicate no statistical difference. For the detection of EVs in crude mixture (Section 3.6), 1-way ANOVA was used with Tukey’s multiple comparison test. They were applied to evaluate the obtained results using GraphPad Prism software version 10.6. Written details on experimental procedures have been submitted to the EV-TRACK knowledgebase (EV-TRACK ID: EV250115).

## 3. Results

### 3.1. Establishing Standardized SPR Label-Free Bioassay for EV Detection Using rEVs

To establish a robust label-free SPR bioassay for EV detection, we first used rEVs (produced from HEK293T cells [39]), comparable to previous studies [40]. A planar C1 sensor chip was used as the backbone for the new immunoassay because it lacks a carboxymethylated dextran matrix, which is present on three-dimensional sensor chips such as CM5 and CM3. This matrix is about 100 nm thick and could prevent EVs from entering the surface layer, potentially hindering their detection. First, an RAM IgG antibody was covalently immobilized on the four individual flow cells of a C1 sensor chip. Next, different mouse antibodies, all EV-specific (i.e., anti-CD9, anti-CD63 and anti-CD81) and anti-CXCR4, were captured on this RAM immunosensor, followed by injection of EV samples (Figure 1). A reference flow cell (Fc 1) without captured antibodies was used to subtract aspecific binding. Buffer blanks were used to correct for baseline drift of the captured mouse antibodies. BSA was added to the buffer to minimize aspecific binding of EVs to the sensor chip and microfluidics. The amount of bound EVs (in RU) was measured 100 s after injection to ensure a stable RU level for accurate measurement. Finally, the chip was regenerated using glycine-HCl pH 1.7 and 0.5% Triton X-100 solution to prepare for capturing new mouse antibodies.

The LOD, specificity and repeatability of the SPR biosensor were first assessed using rEVs. rEVs contain the major structural component of HIV-1 virus particles (the gag polyprotein) fused with the fluorescent protein EGFP and carry the common EV biomarkers (CD9, CD63 and CD81) on their membranes [40,41]. Thus, they are valuable tools as reference materials for method development and data normalization.

**Figure 2:**
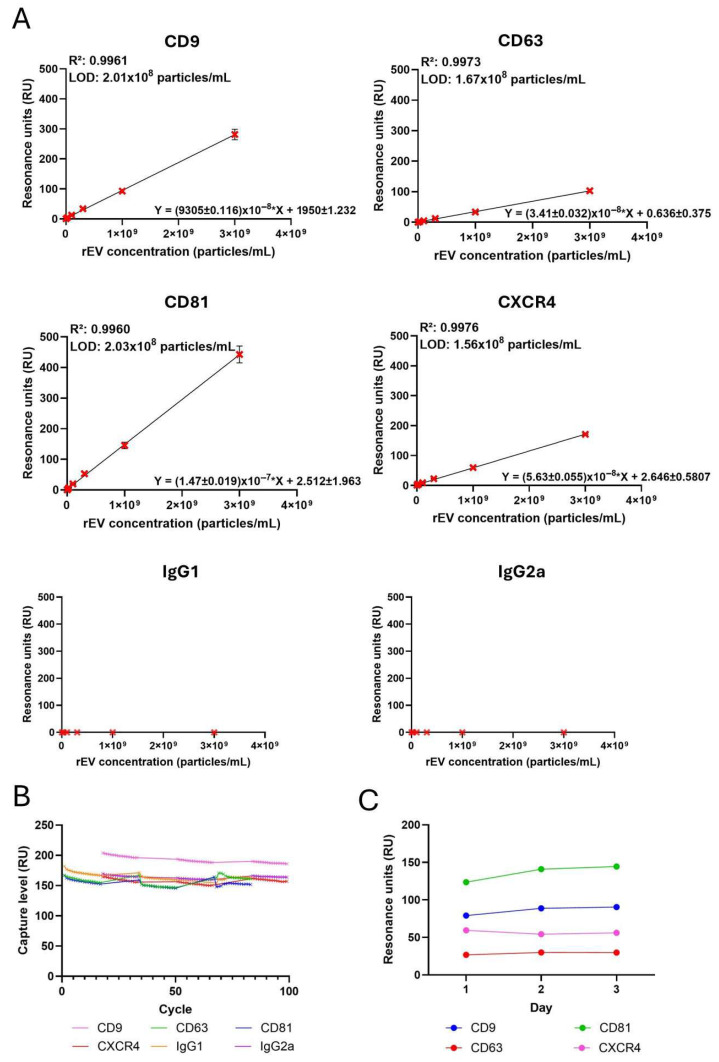
Establishing a standardized SPR label-free bioassay for EV detection using rEVs. (**A**) The calibration curves using a series of rEV concentrations and anti-CD9, anti-CD63, anti-CD81 and anti-CXCR4 antibodies. The RUs were plotted as a function of the rEV concentrations (particles/mL). Simple linear regression was fitted using GraphPad prism software version 10.6. The LOD was calculated using (3.3*σ)/S = LOD, with σ being the standard deviation of the curve and S the slope of the curve. The error bars represent standard deviations (n = 3). (**B**) Stability and repeatability of the chip with different mouse antibodies. The capture levels are stable for at least 100 cycles. (**C**) Binding response from the three measurements of rEVs at a concentration of 1 × 10^9^ particles/mL, illustrating interday precision.

To determine the LOD of the newly established biosensor, different calibration curves were generated for anti-CD9, anti-CD63, anti-CD81, anti-CXCR4 and their respective isotypes using rEVs at concentrations ranging from 1 × 10^7^ to 3 × 10^9^ particles/mL. The obtained average responses (*n* = 3) were plotted as a function of the rEV concentration (Figure 2), whereas the calibration curves were fitted and LOD calculated as explained in Section 2.10. The slopes and intercepts of the antibody calibration curves are shown in Table S2. For anti-CD9, anti-CD63 and anti-CD81, the intercepts were not significantly different from zero, indicating a negligible baseline signal. The intercept of anti-CXCR4 was significantly different from zero, suggesting a small non-zero baseline signal. The obtained LOD values for anti-CD9, anti-CD63, anti-CD81 and anti-CXCR4 are respectively 2.01 × 10^8^, 1.67 × 10^8^, 2.03 × 10^8^ and 1.56 × 10^8^ particles/mL (Figure 2A), all being at least 100 times lower compared to the typical physiological concentration of EVs in human plasma [40]. As the biosensor chip can be regenerated and reused for measurement of different mouse antibodies, the stability and repeatability of the chip was assessed. Figure 2B shows that the SPR biosensor gives a stable and repeatable result for at least 100 cycles. The mean capture levels for all the antibodies, including associated errors and CV, are shown in Table S3. Figure 2C highlights the interday precision, showing three measurements of rEVs at a concentration of 1 × 10^9^ particles/mL performed over multiple days. Figure S2 shows the baseline capture level stability, which remains stable for at least 100 cycles. Interestingly, these results indicate that CXCR4 is also present on the rEVs. Importantly, there was no binding of the EVs to both the isotypes (anti-IgG1 and anti-IgG2a) that were used as negative controls.

### 3.2. Optimization of EV Isolation Protocol Using rEVs

EV isolation processes are labour-intensive, and the yield is often low [41]. In order to develop a robust method for isolating the EVs from different cell lines and to increase the EV yield, an SEC isolation procedure adapted from Boïng et al. [42] was further optimized using rEVs as a reference material (Figure S1). The rEVs were initially spiked in PBS and isolated with SEC to verify the fractions in which the particles will elute. Additionally, ultrafiltration steps were performed before and after SEC isolation to compensate for the dilution effect caused by SEC.

**Figure 3:**
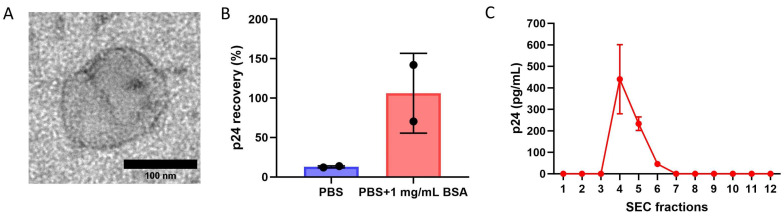
Optimization of EV isolation protocol using rEVs. (**A**) Representative TEM image of rEV. (**B**) Recovery analysis of rEVs using p24 HIV-1 Ag ELISA assay comparing rEVs concentrated in PBS vs. PBS supplemented with 1 mg/mL BSA. Error bars represent standard deviations (n = 2). (**C**) p24 HIV-1 Ag ELISA analysis of all 12 SEC fractions of rEVs. Error bars represent standard deviations (n = 2).

The rEVs, concentrated by ultrafiltration, were first analyzed by TEM to assess their morphological quality. The TEM image shows that their size and round-shaped morphology were as expected (Figure 3A). In order to check ultrafiltration recovery, the rEVs were diluted in two different buffers, PBS and PBS supplemented with 1 mg/mL BSA. The p24 HIV-1 Ag ELISA assay was used to determine the rEV concentrations. rEVs diluted in PBS buffer showed minimal recovery of about 12% after the final ultrafiltration step (Figure 3B). Interestingly, when BSA was added to PBS, the recovery of rEV increased to almost 100% (Figure 3B). Moreover, as presented in Figure 3C, SEC fractions 4 and 5 contained the largest number of rEVs.

### 3.3. Flow Cytometry Analysis of CXCR4 Expression on the Selected Cell Lines

**Figure 4:**
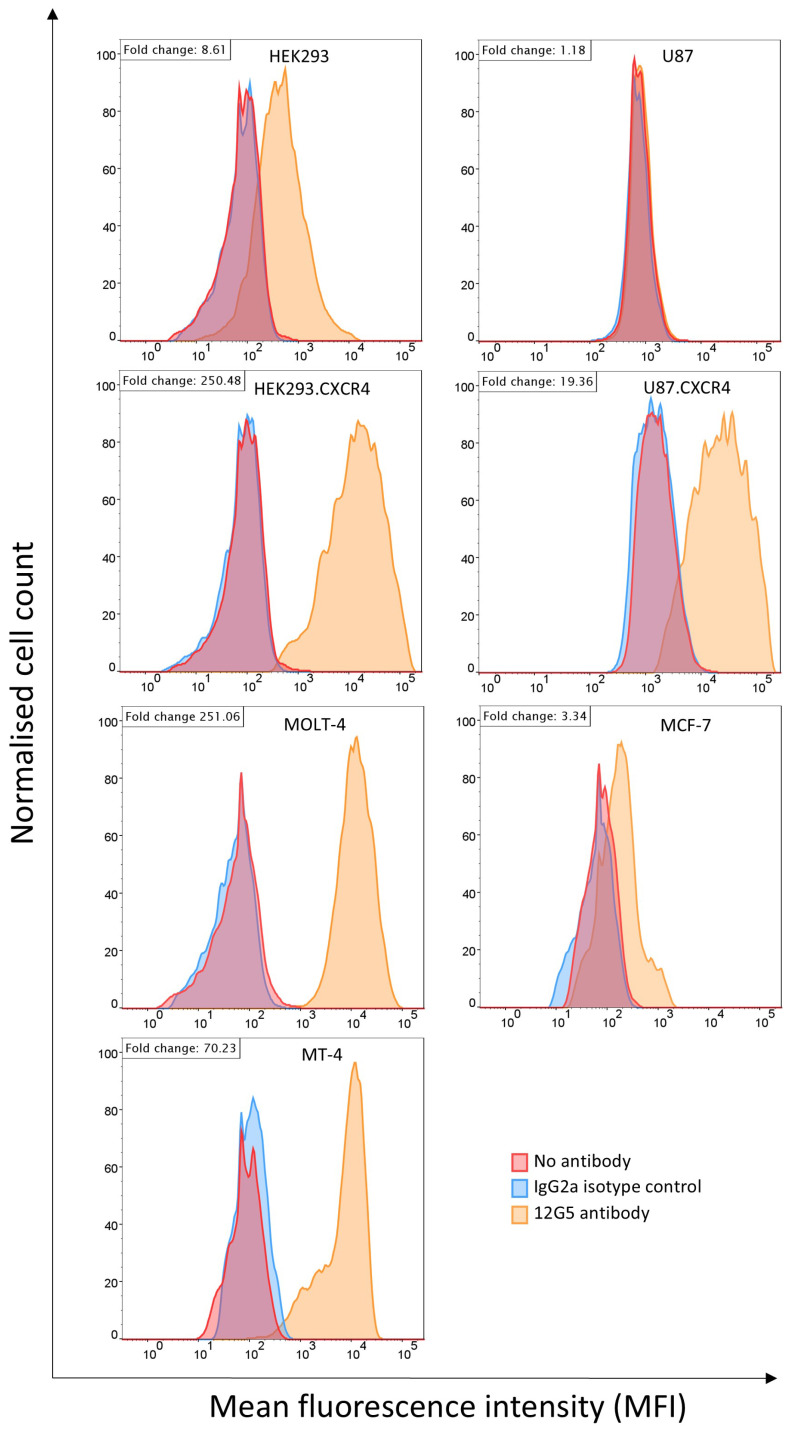
Expression levels of CXCR4 in diverse cell lines. The FCM results of the HEK293, HEK293.CXCR4, U87, U87.CXCR4, MOLT-4, MCF-7 and MT-4 cells. The cells were stained with anti-CXCR4 monoclonal antibody clone 12G5-PE (for U87 and U87.CXCR4 cells) or 12G5-APC (all other cell lines) (orange). IgG2a-PE (for U87 and U87.CXCR4 cells) or IgG2a-APC (all other cell lines) isotype control antibodies were used as the negative control (blue). The cells not stained with an antibody were used as the negative control (red). The fold change was calculated using MFI in the equation (MFI_positive_/MFI_negative_). The negative control is used for MFI_negative_.

Prior to the isolation of EVs, cell surface expression of CXCR4 was measured on seven different cell lines. FCM revealed that all the cell lines express CXCR4, but to a different extent (Figure 4). CXCR4 expression was quantified using fold change by dividing the MFI of the sample by the MFI of the blank. The level of endogenous CXCR4 expression was high in the MOLT-4 and MT-4 cells, with fold changes of respectively 251 and 70. The expression levels in these cell lines were comparable to the level of CXCR4 expression observed in the transfected HEK293.CXCR4 and U87.CXCR4 cells, with fold changes of respectively 250 and 19. In contrast, the non-transfected HEK293 and MCF-7 cells exhibited low-level CXCR4 membrane expression, with fold changes of respectively nine and three. The non-transfected U87 cells showed no CXCR4 expression on their membrane, thus had a fold change of one.

### 3.4. Label-Free Detection of EVs from Different Cell Lines with SPR Bioassay

After examining the CXCR4 expression levels on seven different cell lines, EVs were isolated from all of them using the optimized EV isolation protocol. Using the SPR bioassay, all the SEC fractions where analyzed to identify the fraction with the highest EV concentration (Figure S3). Together with the NTA analysis, which was performed to determine the size and concentration distribution of the isolated EVs, SEC fraction 5 was selected (Figure S4). Notably, the EVs from all the different cell lines had comparable size ranges (170–200 nm) and concentration values (around 10^10^ particles/mL). TEM analysis also confirmed the presence of vesicle structures in a cup-shaped form, indicating EVs presence in SEC fraction 5 (Figure S5).

**Figure 5:**
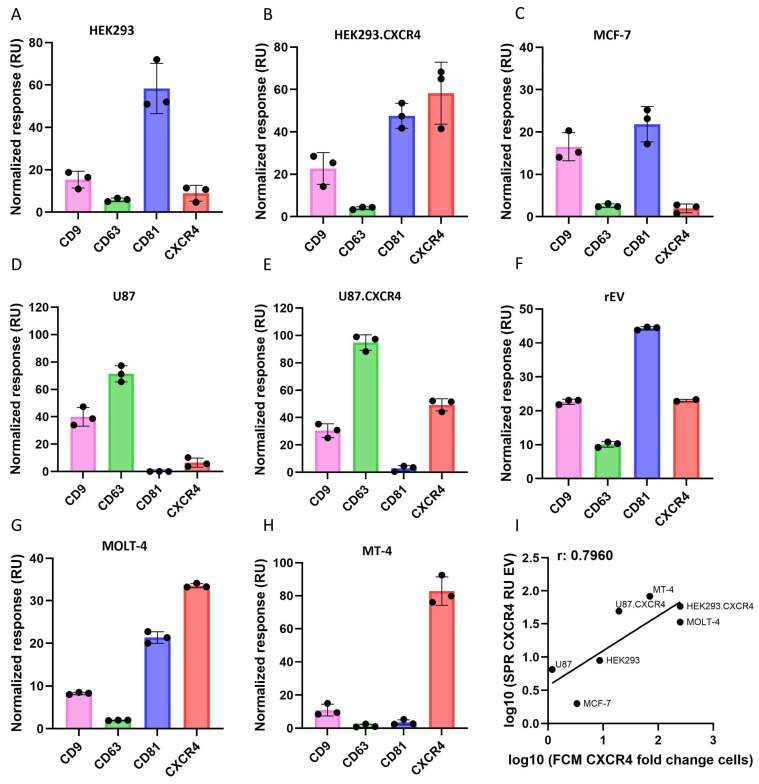
(**A**–**H**) SPR detection of tetraspanins CD9, CD63, CD81 and CXCR4 on purified EVs (SEC fraction 5). EVs were injected over C1 immunosensor functionalized with anti-mouse antibodies onto which anti-CD9, anti-CD63, anti-CD81 and anti-CXCR4 antibodies were captured (as depicted in Figure 1). Binding levels (RU) were normalized based on particle number, as explained in Section 2.9. Error bars represent standard deviations (n = 3). (**I**) Correlation of FCM data on cells and SPR data on EVs. FCM fold change data on cells is correlated with normalized data and is shown as logarithmic curve using Pearson’s correlation analysis, with r = 0.7960 (*p* = 0.032) and 95% confidence interval (0.11–0.97).

Next, the isolated EVs were detected on an SPR platform in a label-free manner using the tetraspanin antibodies (anti-CD9, anti-CD63 and anti-CD81) as well as the anti-CXCR4 antibody, resulting in their unique SPR-MFP (Figure 5A–H). All the results were normalized to RU/10^9^ EVs by dividing the response by the EV concentration values obtained from the NTA experiments. Based on this data it can be concluded that not all the EVs contained similar levels of the CD9, CD63 and CD81 biomarkers (Figure 5A–H), although these tetraspanins are known as universal EV biomarkers often used to indicate the presence of EVs. The rEVs, used for optimizing the isolation procedure, showed to contain all the biomarkers, including CXCR4 (Figure 5F). Their biomarker expression profile is similar to that of HEK293 EVs, as expected, given that the rEVs originate from HEK293T cells. HEK293 and HEK293.CXCR4 EVs mostly expressed CD81, to a lesser extent CD9, and relatively low amounts of CD63 (Figure 5A,B). The MCF-7 EVs contained all three assessed tetraspanins, with CD81 being the most abundant, followed by CD9 (Figure 5C). CD63 was predominant in the U87 and U87.CXCR4 EVs, followed by CD9, while they seemed to contain almost no CD81 (Figure 5D,E). From the known tetraspanins, MT-4 EVs expressed CD9 and low amounts of CD81 and CD63 (Figure 5H). MOLT-4 had CD81 and low amounts of CD9 and CD63 (Figure 5G).

Similarly, the CXCR4 expression profile was different among the EVs originating from the different cell lines. A clear distinction was seen between the HEK293 and U87 EVs and their CXCR4-transfected counterparts, indicating that protein overexpression on the cell membrane is carried over to the EV membrane. The hematopoietic T cell lines, MOLT-4 and MT-4, characterized by a high-level endogenous CXCR4 expression (Figure 4), contained the most intense CXCR4-EV signal compared to the generic tetraspanin markers. Of note, the FCM data of CXCR4 expressed on the membrane of the cell lines (Figure 4) correlated with the SPR data of the CXCR4 expression on the membrane of the EVs of these cell lines (Figure 5I). Pearson’s correlation analysis showed a positive correlation of r = 0.7960 (n = 7 and *p* = 0.032). However the 95% confidence interval was wide (0.11–0.97) due to the small sample size. Importantly, no binding on the IgG1 and IgG2a isotype control antibodies was detected for any of the EVs.

### 3.5. Colocalization of EVs from HEK293 and HEK293.CXCR4 Cells Using SP-IRIS Technology

**Figure 6:**
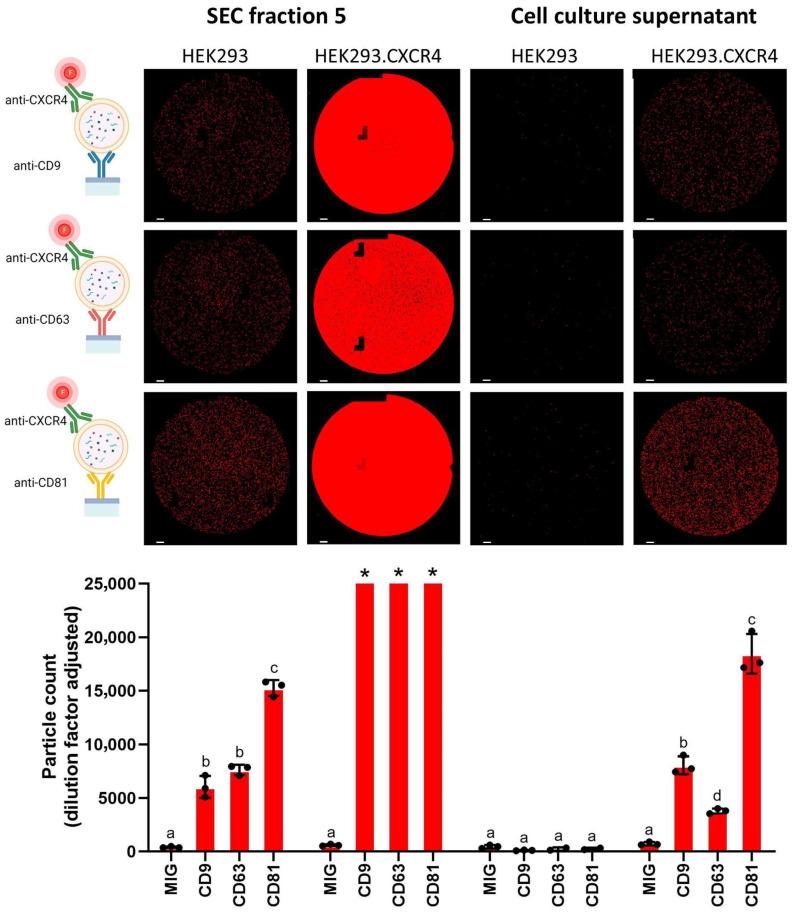
CXCR4 detection on single EVs using SP-IRIS. Colocalization of CXCR4 with CD9, CD63 and CD81 investigated with HEK293 and HEK293.CXCR4 EVs from SEC fraction 5 (**left** panel) and directly in cell culture supernatant (**right** panel). Scale bar = 10 µm. Schematic on left side was created in Biorender.com. Yagmur Yildizhan. (2023). Statistical difference between groups was assessed by mixed effects analysis with Tukey’s multiple comparison test; averages denoted with same letter (a–d) indicate no statistical difference. Out-of-range data points are depicted with asterisk.

To benchmark the SPR results and to visualize the protein expressions of single EVs, the HEK293 and HEK293.CXCR4 EVs were also analyzed using ExoView technology. Figure 6 depicts the number of CXCR4-positive EVs obtained from the ExoView chips, functionalized with anti-CD9, anti-CD63 and anti-CD81 antibodies for their capturing, while detection was performed using the fluorescently labelled anti-CXCR4 antibody. Here, the EVs were detected either from SEC fraction 5, as in the Biacore^TM^ SPR experiments, or directly in the cell culture supernatant (i.e., without any prior purification). NTA results of the different EV samples are shown in Figure S6. All the EV samples were diluted 1:10.

The analysis showed that the CXCR4-harboring EVs from the HEK293.CXCR4 cells are present in both purified CCM (SEC5) and unpurified CCM (Figure 6), with the former having more particles per spot because of the EV enrichment step. However, the channels of the purified EVs from HEK293.CXCR4 were saturated and could not be quantified. The EVs derived from the HEK293 cells, containing CXCR4 in the membrane, could only be detected in the EV-enriched sample (SEC5) and not in the unpurified CCM (no significant difference from the isotype control). The latter was confirmed with the NTA analysis of CCM that showed very few particles in the supernatant.

### 3.6. Detection of EVs Directly in Crude Mixture Using the SPR Biosensor

**Figure 7:**
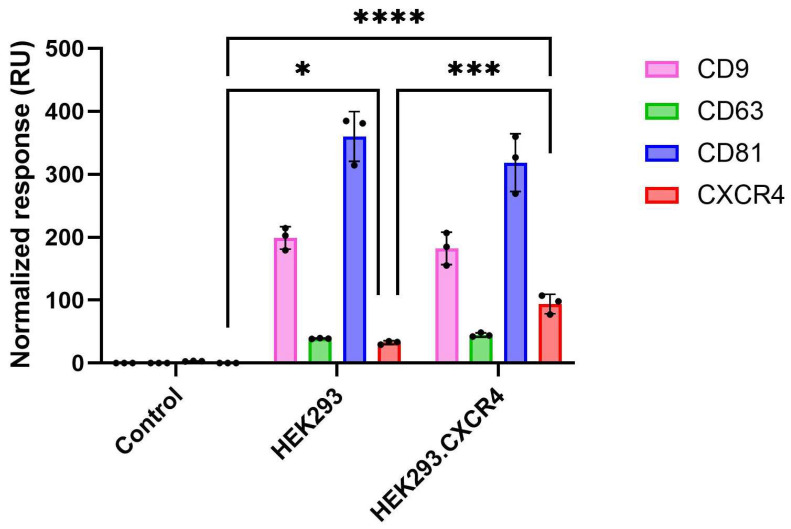
Detection of EVs in crude mixture using Biacore^TM^ SPR technology. Normalized binding levels of HEK293 and HEK293.CXCR4 EVs spiked in DMEM cell medium supplemented with 10% ED-FBS when using anti- CD9, anti-CD63, anti-CD81 or anti-CXCR4 as capture antibodies. DMEM cell medium supplemented with 10% ED-FBS was used as control. Binding levels (RU) were normalized based on particle number, as explained in Section 2.9. Error bars represent standard deviations (n = 3). Statistical difference between groups was assessed by 1-way ANOVA with Tukey’s multiple comparison test (alfa = 0.05) (*: *p* = 0.0109, ***: *p* = 0.0004 and ****: *p* < 0.0001).

As seen in the previous section, Exoview can be used to study EVs in cell culture supernatant, but requires colocalization of two EV protein biomarkers to do so. Therefore, the SPR biosensor was tested to detect EVs directly in crude mixtures in a label-free format without the necessity of colocalization. The EVs isolated from CCM of HEK293 and HEK293.CXCR4 were spiked in a DMEM cell medium containing 10% ED-FBS with a final dilution of 1:10. Normalization was based on concentration values obtained from the NTA experiments. The SPR analysis revealed the presence of three tetraspanins (CD9, CD63 and CD81), as well as CXCR4 (Figure 7). These results were comparable to those obtained in buffer alone (Figure 5A,B), confirming that the EVs from the HEK293 and HEK293.CXCR4 cell lines primarily contain CD81, a lesser amount of CD9 and minimal CD63. Also, the overexpression of CXCR4 in the HEK293.CXCR4 EVs was evident in this experiment with a similar fold increase compared to HEK293, as seen in Figure 5A,B.

## 4. Discussion

In this study, we developed a highly sensitive, label-free EV detection bioassay using Biacore^TM^ SPR technology, with a focus on the specific detection of conformation-dependent CXCR4 on EVs isolated from seven different cell lines. SPR has already proved to be a valuable tool to studying CXCR4–ligand interactions using solubilized CXCR4 captured on a sensor chip or using immobilized virus-like particles harboring CXCR4 in their membrane [43]. Our detection approach is based on the assay of Willis et al., where the authors captured anti-membrane protein antibodies on a goat anti-mouse IgG antibody-derivatized biosensor. Virus-like particles carrying these conformationally complex proteins are used as soluble probes to measure the membrane–protein interactions [44].

Using rEVs as a reference material, we were able to optimize both the isolation and SPR detection of EVs from CCM with high efficiency (i.e., recovery) and reproducibility. Importantly, specificity was established using immunoaffinity-based biosensors that specifically recognize the generic tetraspanins or CXCR4 present on EVs. These are all important characteristics for EV-based biomarker discovery [45,46,47,48]. In our isolation method the addition of BSA proved to increase the EV yield, while importantly BSA did not show any interference with the SPR results. This is in line with a previous report showing that addition of BSA is beneficial to prevent non-specific binding of molecules and particles onto the sensor chip, the microfluidic system of SPR systems and onto the tube walls during storage [47]. Moreover, addition of BSA is believed to improve the long-term storage of EV samples and stability throughout several freeze–thaw cycles [48]. However, BSA is often not considered in EV analysis as it might interfere with downstream analysis such as proteomics. For EV marker analysis techniques, where BSA is undesirable, samples that contain low amounts of EVs might be wrongfully marked as empty, while SPR could pick up an EV signal due to the increased EV yield, as well as high sensitivity.

Our SPR biosensor demonstrated high sensitivity with an LOD of 1.56–2.03 × 10^8^ rEVs/mL depending on the captured antibody. The reported LODs are at least 100 times lower than the typical physiological concentration of EVs in human plasma [42,49]. The detection limit could even be lowered by increasing the contact time of the EV samples. The sensorgrams were not oversaturated at the highest concentration of rEVs (3 × 10^9^ particles/mL), which translates into a broad dynamic range as opposed to SP-IRIS, where signal saturations on single-use chips might occur at higher EV concentrations (Figure 6). Because SPR only detects binding events within the depth of the evanescent wave (~150 nm from the surface), this technology fills the gap of the other technologies (e.g., NTA, nanoFCM, and SP-IRIS) that fail to detect EVs smaller than 50–70 nm [50]. Indeed, extracellular particles as small as adeno-associated virus (25 nm) and poliovirus (30 nm) can easily be detected using SPR technology [51,52,53,54]. It should be noted that this technology remains bulky, but has great potential to complement the above-mentioned technologies for single EV quantification. In fact, SPR can be seen as a valuable orthogonal technique to rapidly detect and analyze EV surface markers and shortlist new biomarkers [53]. While quartz crystal microbalance (QCM) has been applied successfully for EV characterization and phenotypic subtyping, exploiting combined mass and viscoelastic signals to detect tetraspanin-positive EVs even in complex media such as serum, its signal reflects both the hydrated mass and viscoelastic properties of bound EVs [54,55]. In contrast, SPR measurements rely predominantly on refractive index changes confined to the evanescent field, providing a response that more closely reflects the dry mass of bound EVs and is less affected by layer viscoelasticity. This optical detection principle can simplify quantitative interpretation of binding events and facilitates more robust comparative quantification of specific EV surface markers, such as CXCR4, across both purified and complex samples.

Using the established SPR immunoassay, CXCR4 was successfully detected on the EVs of all seven analyzed cell lines. For the CXCR4 immunosensor, we selected the monoclonal antibody 12G5, which specifically recognizes conformation-dependent CXCR4 and does not bind to debris or disintegrated CXCR4 [43]. Together with the generic tetraspanins, SPR-MFP was created for all the different EVs and showed an EV-specific signature per cell type. Colocalization of CXCR4 and tetraspanins was confirmed with SP-IRIS technology at the single EV level when using EVs from HEK293 cells, which is in line with previous studies demonstrating the value of this platform for EV marker colocalization [56]. However, based on our label-free SPR data, demonstrating that EV expression levels of generic tetraspanin markers might be very different among different cell lines, and hence also too low or even absent on some EVs, care should be taken when detecting EVs using a sandwich approach like on an SP-IRIS platform. Indeed, our SPR results showed no expression of CD63 and CD81 on the isolated EVs from respectively the MOLT-4 and U87 cells. This could lead to false negative results when one of these tetraspanin markers is used as a capture or detection antibody.

It is interesting to note that CXCR4 was found on all the isolated EVs, although the amount was different among the tested cell lines. Recently it was shown that GPCRs play a significant role in the life cycle of EVs and that EVs can attenuate GPCR signaling [17]. Therefore, CXCR4-positive EVs can be seen as an important and new player in the activity regulation of CXCR4 and its ligand CXCL12 [17,57]. The MOLT-4, MT-4 and HEK239.CXCR4 EVs contained the highest amount of CXCR4-positive EVs. This result is in line with the FCM profiles that indicated a high level of expression of CXCR4 on the cell surface. Moreover, a clear distinction was seen between the original cells and their CXCR4-transfected counterparts, indicating that protein overexpression is carried over to the EV membrane. This might have applications in several interesting fields, one of them being SPR kinetic studies with EVs as drug discovery agents immobilized on a sensor chip and biologicals or small molecules free in a solution [45,46]. From these experiments it can be concluded that CXCR4 expression on the cell surface correlates to CXCR4 expression on the EV membrane. Therefore, detecting CXCR4-positive tumors using CXCR4 expression analysis on EVs by SPR can become a promising diagnostic tool.

It should be noted that it is difficult to compare the RU values obtained from antibodies recognizing different epitopes. Because the observed dissociation rate constant (k_off_) on the biosensor is typically close to zero (Figure 1C), the association rate constant (k_on_) is the determining factor of the antibody-EV complex formation [44]. As a result, antibodies with a high k_on_ will bind more EVs (stronger RU responses) during the injection phase before reaching a steady state, and vice versa. Ideally, one can first screen for different capture antibodies in order to maximize the EV binding response. Nevertheless, the same antibody can be used to compare the EV numbers between different cell lines, making it possible to generate an SPR-MFP in a fast and sensitive way. In an attempt to compare the RU values between the different cell lines, we chose to normalize the RU responses using the NTA-derived particle concentration. However, it is important to highlight that particles smaller than 70 nm are not counted by NTA, and consequently will bias the SPR results. Although there is no size limit for SPR, we believe this normalization can be of an added value when EVs are isolated to a monodisperse SEC population.

SPR chips allowed other researchers to perform over 100 analysis cycles without degradation of the surface or loss of signal, as shown previously [44]. The time for analysis using the current set up (one reference and three active flow cells) is approximately 30 min per sample repetition, which supports the analysis of three biomarkers at once. The flexibility to customize the biosensor with a wide variety of antibodies makes it an attractive tool for screening different membrane proteins. In addition, the low sample consumption rate of SPR (5 µL) as well as integrated automated liquid handling with a cooled autosampler (to preserve EV integrity) makes the developed SPR biosensor a promising diagnostic tool in cancer (as well as other pathogenic) research. Interestingly, the possibility to directly detect EVs in crude mixtures with Biacore^TM^ technology not only reduces the total workload, but also greatly reduces the impact of pre-analytical variables, and consequently improves sample quality. Given the successful detection of tetraspanins and CXCR4 on the spiked EVs in the cell culture supernatant supplemented with EV-depleted serum (Figure 7), the next step is to explore the detection of endogenous EVs in plasma samples, where the concentration of EVs is typically outperformed by lipoproteins (VDL, HDL, and VLVL) that co-isolate with EVs and might affect downstream EV analysis. Alternatively, 12G5 can be used as a capture or detection antibody on other SPR platforms, such as a fiber optic (FO)-SPR biosensor. This alternative biosensor has already shown to be a promising tool in detecting EVs in crude mixtures, while offering advantages over microfluidic techniques as it is less prone to clogging and more cost-effective [38].

In conclusion, we developed a fast and robust method to isolate EVs as well as a sensitive, specific and label-free approach to detect and profile EV membrane proteins using SPR. The reference material, rEVs, was used to improve our research’s rigor and standardization, which fulfills the recommendations of MISEV [6]. The high efficiency, sensitivity, specificity and repeatability of the biosensor make it an ideal tool for the first-line screening of EV biomarkers. With this, CXCR4 was discovered to be present on all the isolated EVs and correlated with expression on the parental cell line. Further investigations into the potential of CXCR4 as a general EV biomarker are needed. Additionally, SPR detection of EVs in crude mixtures using Biacore^TM^ technology harbors great potential as a diagnostic tool and will be further explored.

## Data Availability

The original contributions presented in this study are included in the article/Supplementary Material. Further inquiries can be directed to the corresponding authors.

## Supplementary Materials

The following supporting information can be downloaded at: https://www.mdpi.com/article/10.3390/bios16030174/s1, Table S1: Overview of cell lines; Figures S1: Isolation protocol of extracellular vesicles (EVs); Table S2: Overview of the slopes and intercepts of antibody calibration curves; Table S3: Mean capture levels, corresponding errors, and coefficients of variation (CV) for all antibodies; Figure S2: Baseline capture level stability of the SPR biosensor; Figure S3: SPR detection of CD9, CD63 and CD81 on EV enriched SEC fractions; Figure S4: Nanoparticle tracking analysis results of isolated EVs; Figure S5: Characterization of EVs’ morphology; Figure S6: Nanoparticle tracking analysis results of HEK293 and HEK293.CXCR4 EVs.
