# Supplementary material for: Surface Plasmon Resonance as a Potential Diagnostic Tool for the Detection of CXC Chemokine Receptor 4 (CXCR4) on Extracellular Vesicles
Source: Biosensors (Basel). 2026 Mar 21;16(3):174. doi: 10.3390/bios16030174 (PMC13024074; doi:10.3390/bios16030174)
Supplement: Supplementary file 1 [file biosensors-16-00174-s001.zip › biosensors-4181197-supplementary.pdf]

biosensors

MDPI

Supplementary Materials

Surface Plasmon Resonance as a Potential Diagnostic Tool for the Detection of CXC Chemokine Receptor 4 (CXCR4) on Extracellular Vesicles

Kaat Verleye [1,*,†], Sam Noppen [1,†], Arnaud Boonen [1], Yagmur Yildizhan [1,2], Tom Van Loy [1], Cindy Heens [3], Frank Vanderhoydonc [4], Cláudio Pinheiro [5], Paula M. Pincela Lins [6], Annelies Bronckaers [6], An Hendrix [5], Johannes V. Swinnen [4], Dragana Spasic [2], Jeroen Lammertyn [2], Christophe Pannecouque [3] and Dominique Schols [1,*]

[1] Molecular, Structural and Translational Virology (Rega Institute), Department of Microbiology, Immunology and Transplantation, KU Leuven, 3000 Leuven, Belgium
[2] Biosensors Group, Department of Biosystems, KU Leuven, 3000 Leuven, Belgium
[3] Molecular Genetics and Therapeutics in Virology and Oncology (Rega Institute), Department of Microbiology, Immunology and Transplantation, KU Leuven, 3000 Leuven, Belgium
[4] Laboratory of Lipid Metabolism and Cancer, Department of Oncology, KU Leuven, 3000 Leuven, Belgium
[5] Laboratory of Experimental Cancer Research, Department of Human Structure and Repair, Ghent University, 9000 Ghent, Belgium
[6] Department of Cardio and Organ Systems (COS), Biomedical Research Institute, Hasselt University, 3590 Diepenbeek, Belgium
* Correspondence: kaat.verleye@kuleuven.be (K.V.); dominique.schols@kuleuven.be (D.S.)
† These authors contributed equal to this work.

 https://doi.org/10.3390/bios16030174

Table S1. Overview of the cell lines. The table indicates the used cell lines, cell culture medium (CCM) during the first 72 h and the origin of the cell lines.

Cell line	CCM	Source
HEK293	DMEM + 10% FBS	Provided by Dr. A. Inoue (Tohoku University, Sendai, Japan)
HEK293.CXCR4	DMEM + 10% FBS + 500 µg/mL geneticin	Provided by Dr. A. Inoue (Tohoku University, Sendai, Japan)
U87	DMEM + 10% FBS + 1% HEPES + 1 mM sodium pyruvate	Provided by Dr. D. R. Littman (Skirball Institute of Biomolecular Medicine, NY, USA)
U87.CXCR4	DMEM + 10% FBS + 1% HEPES + 1 mM sodium pyruvate + 500 µg/mL geneticin	Provided by Dr. D. R. Littman (Skirball Institute of Biomolecular Medicine, NY, USA)
MOLT-4	RPMI 1640 + 10% FBS	ATCC
MCF-7	DMEM + 10% FBS + 1% HEPES + 1 mM sodium pyruvate	ATCC
MT-4	RPMI 1640 + 10% FBS	Provided by Prof. L. Montagnier (Pasteur Institute, Paris, France)

https://doi.org/10.3390/bios16030174

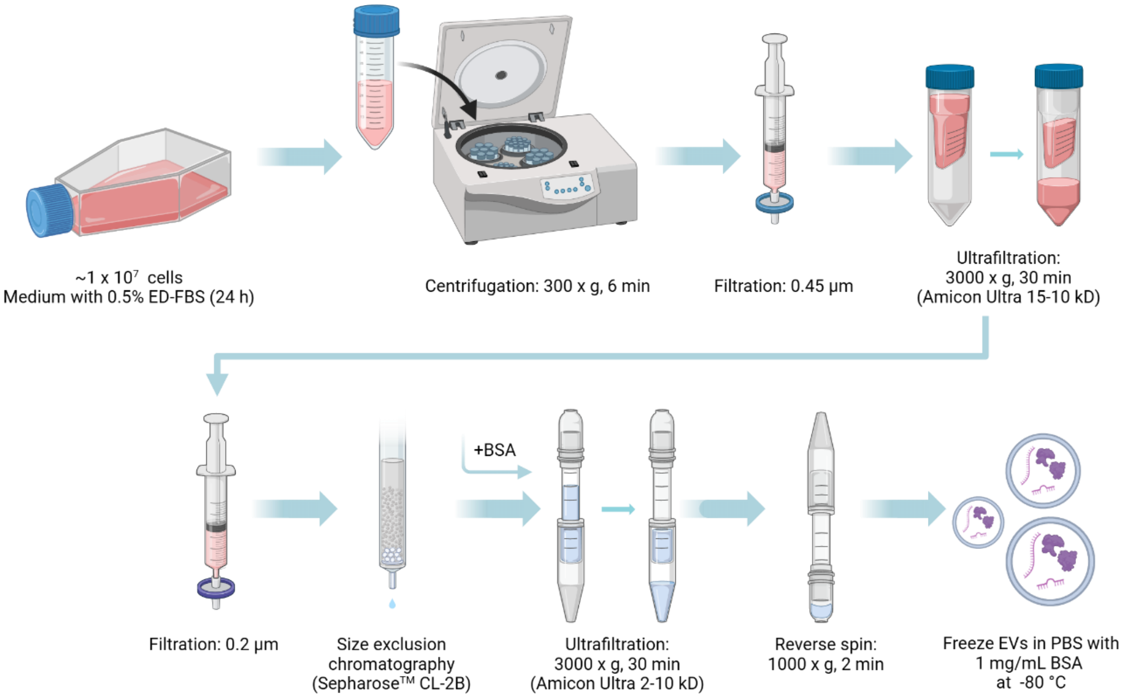

Figure S1. Isolation protocol of extracellular vesicles (EVs). Schematics of SEC EV isolation procedure starting from two T75 flasks of HEK293, HEK293.CXCR4, U87, U87.CXCR4, MOLT-4, MCF-7 and MT-4 cells. Created with BioRender.com.

https://doi.org/10.3390/bios16030174

Table S2. Overview of the slopes and intercepts of antibody calibration curves. The table shows the slope and intercept values together with their 95% confidence intervals (CI).

Antibody	Slope (RU × mL/particles)	95% CI slope (RU × mL/particles)	Intercept (RU)	95% CI intercept (RU)
Anti-CD9	9.31×10^{-8}	9.07×10^{-8}–9.55×10^{-8}	1.95	−0.59–4.79
Anti-CD63	3.41×10^{-8}	3.33×10^{-8}–3.48×10^{-8}	0.64	−0.14–1.41
Anti-CD81	1.47×10^{-7}	1.43×10^{-7}–1.50×10^{-7}	2.51	−1.53–6.56
Anti-CXCR4	5.63×10^{-8}	5.52×10^{-8}–5.75×10^{-8}	2.65	1.45–3.84

https://doi.org/10.3390/bios16030174

Table S3. Mean capture levels, corresponding errors, and coefficients of variation (CV) for all antibodies.

Antibody	Mean capture level (RU)	CV (%)
Anti-CD9	192.55 ± 5.24	2.72
Anti-CD63	157.76 ± 7.09	4.49
Anti-CD81	153.02 ± 4.83	3.16
Anti-CXCR4	157.26 ± 3.83	2.43
IgG1	165.35 ± 5.28	3.20
IgG2a	163.92 ± 2.55	1.55

https://doi.org/10.3390/bios16030174

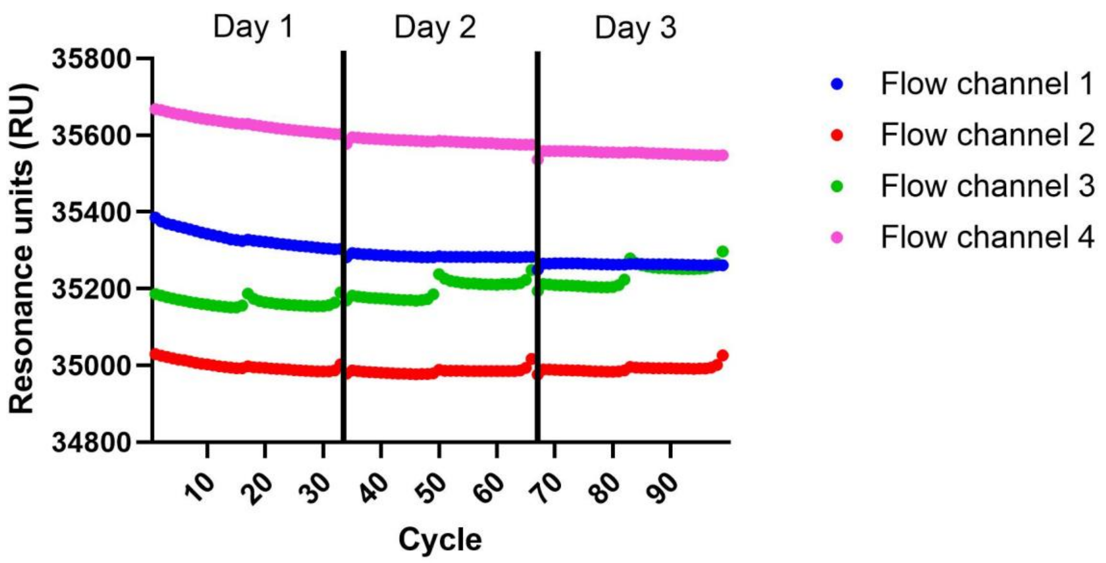

Figure S2. Baseline capture level stability of the SPR biosensor. The baseline capture level remains stable for at least 100 cycli. Measurements were performed over three days to demonstrate both intra- and interday stability.

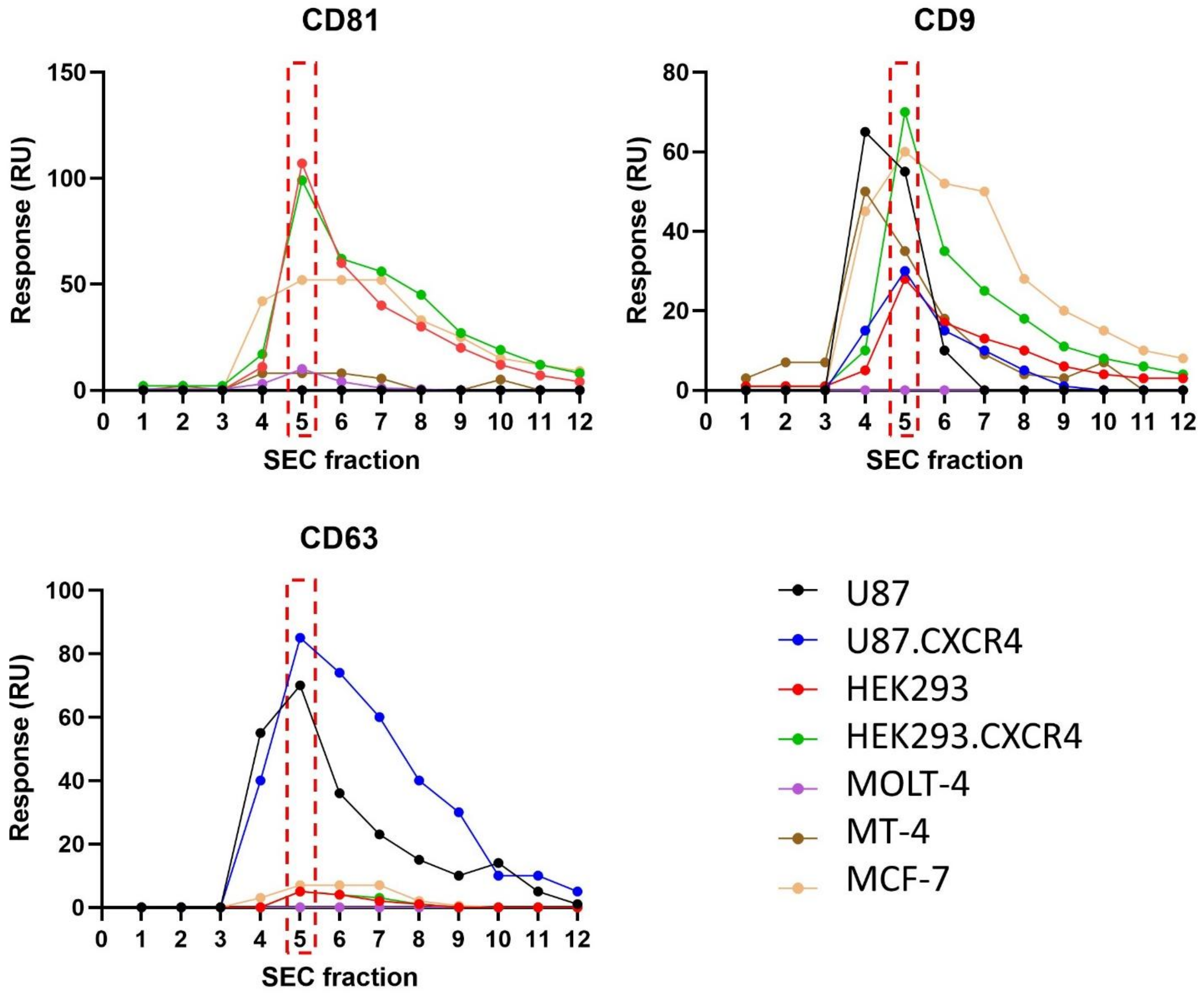

Figure S3. SPR detection of CD9, CD63 and CD81 on EV enriched SEC fractions. The fractions were obtained from SEC for the EVs of HEK293, HEK293.CXCR4, U87, U87.CXCR4, MOLT-4, MCF-7 and MT-4 cells. All fractions were diluted 1/10 and injected over a C1 sensor chip functionalized with anti-CD9, anti-CD63 and anti-CD81.

https://doi.org/10.3390/bios16030174

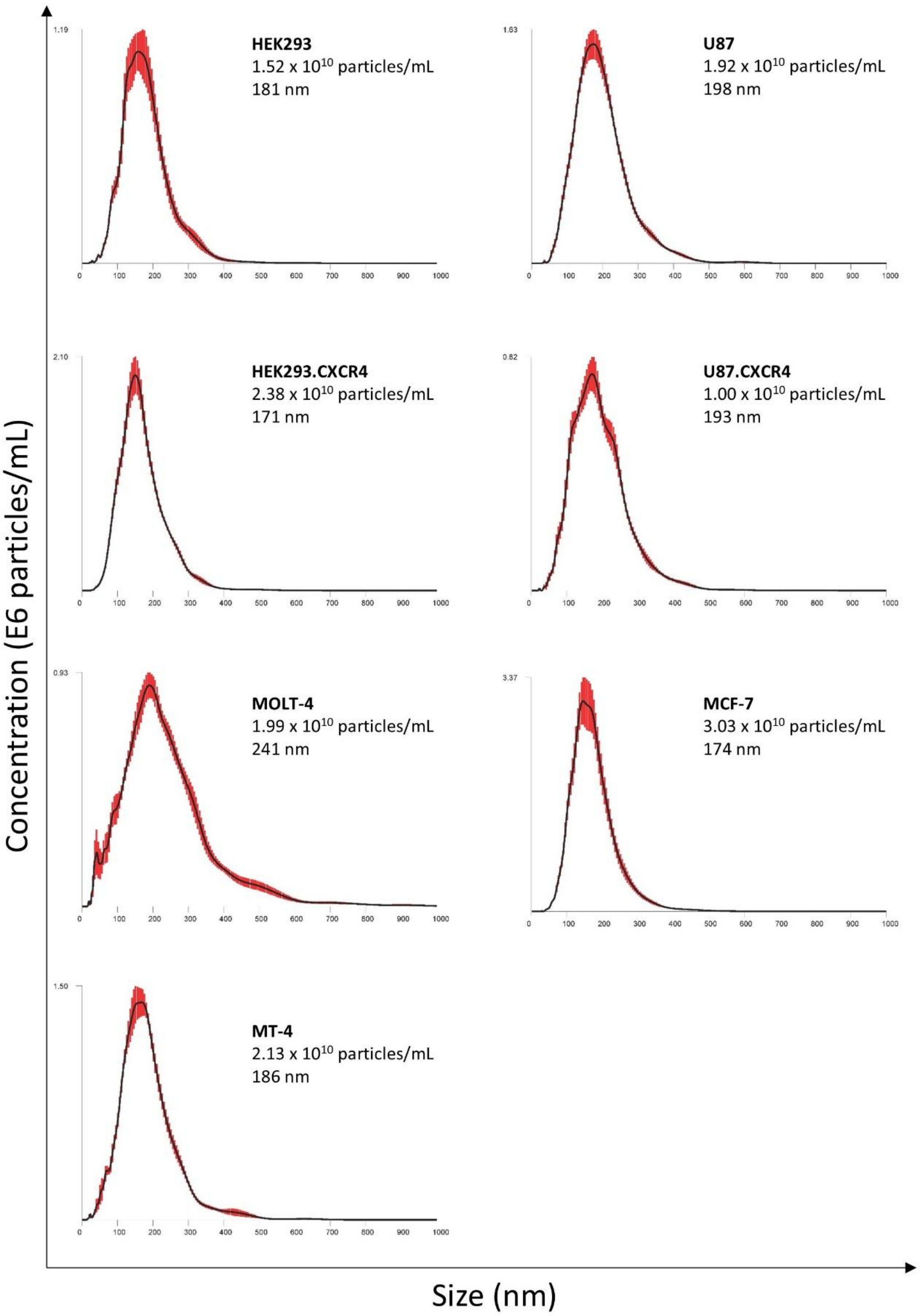

Figure S4. Nanoparticle tracking analysis results of isolated EVs. Representative nanoparticle tracking analysis of EVs from SEC fraction 5 derived from HEK293, HEK293.CXCR4, U87, U87.CXCR4, MOLT-4, MCF-7 and MT-4 cells. Particle size distribution is given, and particle size and amount were calculated and listed on each graph.

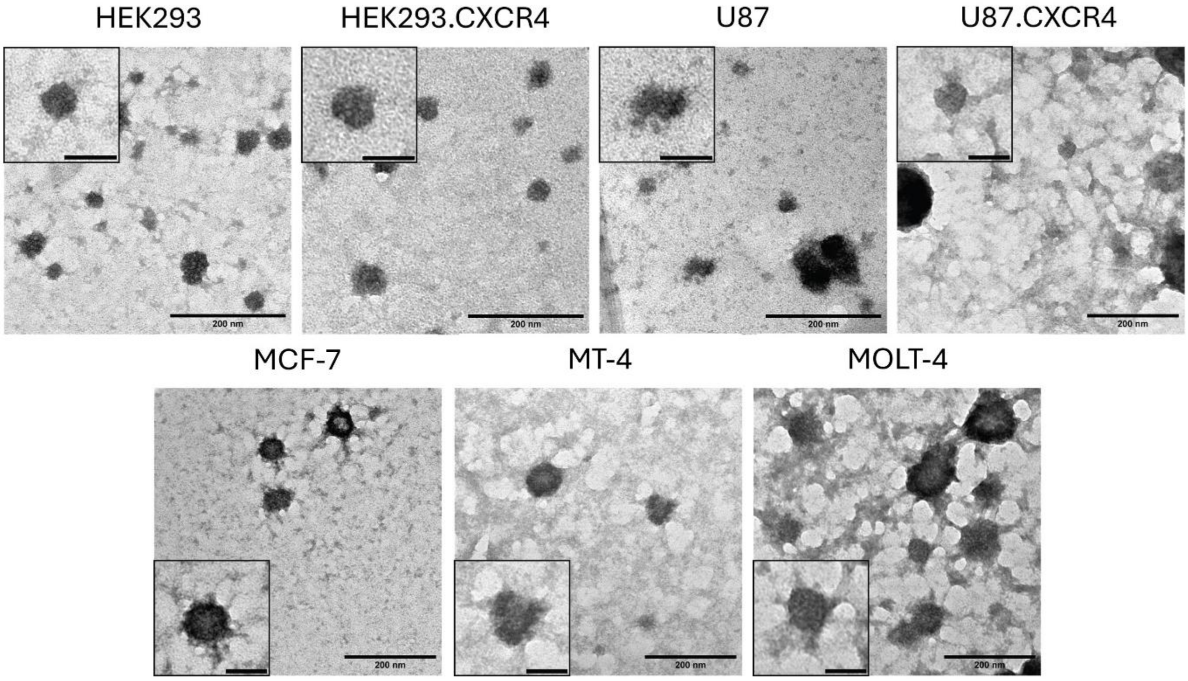

Figure S5. Characterization of EVs' morphology. Representative transmission electron microscopy images of EVs isolated from HEK293, HEK293.CXCR4, U87, U87.CXCR4, MCF-7, MT-4 and MOLT-4 cells. Scale bar overview: 200 nm; insert: 50 nm.

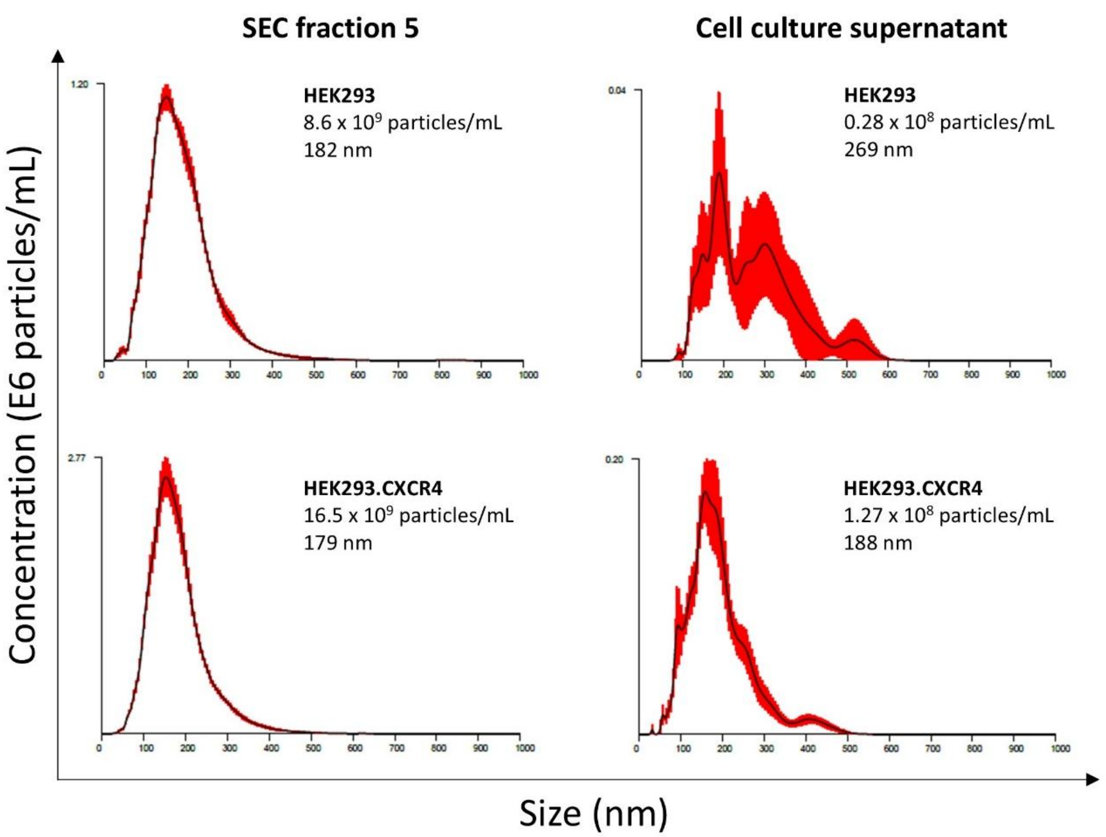

Figure S6. Nanoparticle tracking analysis results of HEK293 and HEK293.CXCR4 EVs. Representative nanoparticle tracking analysis of EVs from SEC fraction 5 and cell culture supernatant derived from HEK293 and HEK293.CXCR4 cells. These EVs were used in the colocalization experiments using Exoview technology. Particle size distribution is given, and particle size and amount were calculated and listed on each graph.

https://doi.org/10.3390/bios16030174
